# Determinants of postnatal care utilization in urban community among women in Debre Birhan Town, Northern Shewa, Ethiopia

**DOI:** 10.1186/s41043-018-0140-6

**Published:** 2018-04-19

**Authors:** Banchalem Nega Angore, Efrata Girma Tufa, Fithamlak Solomon Bisetegen

**Affiliations:** 1 Department of Midwifery, College of Heath Science and Medicine, Wolaita Sodo University, Wolaita Sodo, Ethiopia; 2 School of Public Health, Department of Human Nutrition and Reproductive Health, College of Health Sciences and Medicine, Wolaita Sodo University, Wolaita Sodo, Ethiopia; 3 Department of Medical Laboratory, College of Health Sciences and Medicine, Wolaita Sodo University, Po.box 138, Wolaita Sodo, Ethiopia; 4 Department of Medical Laboratory, Wolaita Sodo University teaching referral Hospital, Wolaita Sodo, Ethiopia; 5 School of Medicine, College of health science and Medicine, Wolaita Sodo University, Wolaita Sodo, Ethiopia

**Keywords:** Women, Postnatal care utilization, Determinants, Debre Birhan, Ethiopia

## Abstract

**Background:**

Reducing maternal mortality and improving maternal health care through increased utilization of postnatal care utilization is a global and local priority. However studies that have been carried out in Ethiopia regarding determinants are limited. So This study aims to assess the magnitude of postnatal care utilization and its determinants in Debre Birhan Town, North Ethiopia.

**Methods:**

A community-based cross-sectional study was conducted from March 1 to April 25, 2015, in Debre Birhan Town. Data were collected through face-to-face interviews using structured pre-tested questionnaires. The data were entered and cleaned in Epi Info version 3.5 and analyzed using SPSS version 20. Bivariate and multiple logistic regression analyses were used. Variable with *p* value less than or equal to 0.2 at bivariate analysis were entered into multiple logistic regression. Significance was declared at 0.05 in multiple logistic regressions and considered to be an independent factor.

**Result:**

From the total respondents, we found that 327 (83.3%) mothers utilized the postnatal care services. Single mothers were less likely to utilize postnatal care services than those mothers who are married and live together [adjusted odds ratio (AOR) = 0.06, 95% CI (0.01, 0.45)]. This study revealed that respondent’s knowledge about postnatal care services is an important predictor of postnatal care utilization [AOR = 0.03, 95% CI (0.00, 0.44)] and mothers who delivered in a health care facility were more likely to receive PNC than mothers who did not deliver in a health care facility [AOR = 0.65, 95% CI (0.58, 0.94)].

**Conclusion:**

The postnatal care utilization rate in Debre Birhan town was 83.3%. Marital status, maternal knowledge, and place of delivery were predictors of postnatal care service utilization. So specific attention should be directed towards the improvement of women’s education since the perception of the need for PNC services were positively correlated with the mother’s education.

## Background

According to WHO, the postnatal, also called postpartum, is the period begins from 1-h after the delivery of the placenta and continues until the 6 weeks (42 days) [1]. Postnatal care (PNC) is regarded as the most important maternal health care service for the prevention of physical and cognitive impairments as well as disability resulting from a postnatal causes [2, 3]. Care during this period is critical for the health and survival of both the mother and the newborn [4].

In 2015, more than 300 million women suffered from pregnancy-related complications and disabilities. Eight hundred thirty women also died each day from pregnancy-related complications [5]. In the year 2015, it was estimated that roughly 303,000 women died due to pregnancy-related complication in low-resource settings [6]. As a result of inadequate postnatal care services, more than two thirds of maternal and newborn deaths occur. The majority of maternal deaths (62%) occur in the postnatal period and more than half of these takes place within a day of delivery [7].

In Ethiopia, maternal mortality ratio (MMR) in Ethiopia is 676 per 100,000 which is significantly higher than the developing country rate and the global rate. The MMR of Ethiopia contrasts with that of UK and the global average, which is 12 and 400, respectively. The national average of the postnatal coverage is only 8% [8]. Compared to antenatal care and skilled attendance at birth, the postnatal care in Ethiopia has been described as a neglected area of maternity care even in safe motherhood programs in Ethiopia [9].

The risk of maternal death is highest close to birth; it then decreases over the subsequent days, weeks and months. Of postpartum deaths, 45% occur within 1 day of delivery, more than 65% within 1 week, and in excess of 80% within 2 weeks [10].

Studies indicated that the extent of PNC services utilization was associated with factors like maternal age, educational level, occupation, place and mode of delivery, number of pregnancies, awareness about obstetric-related danger sign, and awareness about PNC services. The determinants of utilization of PNC services are not also the same across different cultures and socioeconomic status within a society [11–16].

Even though most of the maternal deaths happen during postpartum period, in developing countries, there are a small number of women turning up for PNC than ante-partum and intra-partum care [17].

The impact of the low coverage of postnatal care in Ethiopia is reflected as high neonatal deaths, as well as maternal morbidity and mortality. As a result, the nation did not achieve the millennium development goals (MDGs) that are related to child and maternal mortality (MDG 4 and MDG 5) [18]. The low PNC coverage is a challenge that needs to be addressed [19].

As per the 2015 Ethiopia Demographic and Health Survey (EDHS), Debre Birhan town was a model city for Amhara region to improve the postnatal service care and to achieve the MDG goals. As a result, investigating determinants of under-utilization of postnatal services is a worthy endeavor. By investigating the determinants, this study fills the empirical research gap. This study hypothesized that the PNC coverage in Debre Birhan town is higher than the EDHS national coverage.

## Methods

### Study design

A community-based cross-sectional study design was used.

### Study period and area

The study was conducted from March 1 to April 25, 2015, in Debre Birhan town, North Shoa, 130 km North East of Addis Ababa. The town is administratively divided into nine Kebeles. According to the 2007 census report, Debre Birhan town has a total population of 65,214 of which 33,556 are females. Out of the 33,556 females, the estimated number of women of child-bearing age (15–49 years) is 21,792. The town has two hospitals, four health centers, nine health posts, and six pharmacies.

### Eligibility criteria

All mothers who gave birth prior to 6 weeks to 12 months of the survey were included whereas mothers who gave birth less than 6 weeks to data collection period were excluded.

### Sample size and sampling techniques

#### Sample size

The sample size was determined by using a single population proportion formula considering the following assumptions: *P* = 8% taken [9], level of significance to be 95% (*α* = 0.05), *Zα*/2 = 1.96, absolute precision or margin of error to be 4% (*d* = 0.04), and design effect = 2

$$ n={\left(\mathrm{Z}a/2\ \right)}^2\times P\ \left(1-P\right)/{d}^2={(1.96)}^2\times 0.08\times 0.92/{(0.04)}^2\times 2=354 $$

By taking 10% of non-response rate, 390 mothers were included.

#### Sampling procedure

A multistage sampling procedure was used to select from the total of nine Kebeles. Four Kebeles were selected using simple random sampling technique, and in each selected Kebele, the total sample was proportionally distributed to the four Kebeles. Finally, the households from each Kebele were selected using systematic sampling. The sampling frame of the households (owners) were prepared by using the mother list of the health extension workers.

### Data collection tools and techniques

For data collection, a face-to-face interview was performed using a structured pre-tested questionnaire. Four nurses were used to collect data. Two midwives from Debre Birhan University Hospital were assigned to supervise the data collection process. Both the data collectors and supervisors underwent a 2-day intensive training before the actual work about the aim of the study, procedures, data collection techniques, the art of interviewing, ways of collecting the data, and clarification were given.

### Variables of the study

Dependent variables: utilization of postnatal care service.

### Independent variables

- Socio-demographic characteristics: age, marital status, religion, parity, culture, income, educational status, occupation, husband’s education, and husband’s occupation
- Environmental factors: distance from nearby health institutions
- Behavioral/clinical factors: knowledge, history of antenatal care, type of delivery, and place of delivery

### Data quality control

Questionnaire was pre-tested in one of the Keble other than the selected Kebeles on 5% of participants. Every day after data collection, questionnaires were reviewed and checked for completeness and relevance by the supervisors and a principal investigator, and the necessary feedback were offered to data collectors in the next morning.

### Data processing and analyses

Data were entered into Epi Info version 5.3.1 statistical software and then transferred to statistical package for social science (SPSS) windows version 20.0 for further analysis. Bivariate analysis was used primarily to check which independent variables had an association with a *p* value less than (0.25%) that of the dependent variable. Variables found to have association were entered into a multivariate analysis for controlling the possible effect of confounders, and finally, the variables which had a *p* value less than 0.05 were taken as a statistically significant association which was identified on the basis of an OR with 95% CI.

## Results

### Socio-demographic characteristics

A total of 390 mothers were participated in the study. The mean age of the respondents was 28.65 ± 1.35 years, and the age ranges from 15 to 46 years. Concerning marital status, the majority of the respondents 328 (84.1%) were married. Majority of the respondent were Orthodox Christians amounting to 354 (90.6%). Regarding educational status, 162 (41.5%) had attended at least primary school. By ethnic composition, most of the respondents were Amhara 375 (96.2%) (Table 1).

**Table 1:** Socio-demographic characteristics of the study participants in Debre Birhan Town, North Shewa, 2015 (*n* = 390)

Variables		Number	Percent
Religion	Orthodox Christian	354	90.8
	Muslim	22	5.6
	Protestant	11	2.8
	Other	3	0.8
Ethnicity	Amhara	375	96.2
	Oromo	14	3.6
	Other	1	0.25
Marital status	Married	328	84.1
	Unmarried	31	7.9
	Divorced	24	6.2
	Widowed	7	1.8
Educational status	Illiterate	45	11.5
	Grades 1–8	162	41.5
	Grades 9–12	122	31.3
	12+	61	15.6
Occupation status	Housewife	196	50.3
	Self-employed	138	35.4
	Government employee	56	17.1
Husband’ s education	Illiterate	16	4.1
	Grades1–8	119	30.5
	Grades 9–12	52	39
	12+	103	26.4
Family size	1–3	130	33.3
	4–6	231	59.2
	7–10	27	6.5
Average monthly income	< 500	108	27.7
	501–1500	201	51.5
	1501–2500	60	15.4
	> 2501	21(5.4)	

### Obstetrics characteristics of the respondents

The vast majority (365, 93.6%) of the women were pregnant between the age of 18 and 35 years. It is only 23 (5.9%) who were below 18 years of age when they were first pregnant with a mean age at first pregnancy being 23.1 ± 3.9 years. Of all the respondents, 154 (39.5%) women had only one pregnancy. The majority of the women (215, 55.1%) reported that they had two to four pregnancies (Table 2).

**Table 2:** Obstetrics characteristics of study participant in Debre Birhan town, North Shoa, Ethiopia 2015 (*n* = 390)

Variables	Number	Percent
Age at first pregnancy		
< 18	23	5.9
18–35	365	93.6
35 and above	2	.5
Gravidity		
1	154	39.5
2–4	215	55.1
≥ 5	21	5.4
Number of live births		
1	162	41.5
2–4	211	54.1
≥ 5	17	4.4
Ever had abortion		
No	275	96.2
Yes	15	3.9
Ever had still birth		
No	380	97.4
Yes	10	2.6
Ever had neonatal death		
Yes	378	96.9
No	12	3.1

### Knowledge of the respondents

**Fig. 1:**
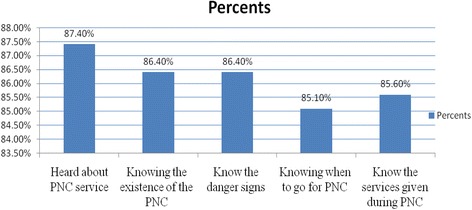
Knowledge of the mothers about PNC in Debre Birhan town, North Shoa, Ethiopia

About 341 (87.4) mothers had heard about PNC services, 337(86.4%) knows the danger signs during postnatal period, and 85.6% of them know the types of services which are given during PNC (Fig. 1). Concerning knowledge about the timing of PNC service, 199 (51%) visited the PNC service 8–42 times, 81 (20.8%) visited 4–7 times, and the rest (52, 13.3%) visited 2–3 times.

### Environmental, clinical, and behavioral factors

Majority of the respondents gave birth at health institutions 354 (90.8%). Three hundred thirty (84.6%) responded the PNC service is easy to get. More than half of the respondents (219, 56.2%) live a distance between 501 and 2000 m from their home to a nearby institution.

A large proportion of the mothers (358, 91.8%) had history of ANC follow-up and 341 (87.4%) were delivered spontaneously. About 306 (78.5%) answered no for presence of cultural barrier for not getting a PNC service (Table 3).

**Table 3:** Environmental, clinical, and behavioral factors of the respondents in Debre Birhan town, North Shoa, Ethiopia 2015 (*n* = 390)

Name of the variable	Frequency	Percent (%)	*p* value
Place of delivery			
Home delivery	36	9.2	0
Institutional delivery	354	90.8	0
Is it easy to get PNC services?	333	85.40%	0
Distance from health institution (meter)			
< 500	92	23.6	0.924
500–2000	219	56.2	0.0000
> 2000	79	20.3	0.0000
ANC follow-up	358	91.8	0.0000
Mode of delivery			0.8570
Spontaneous delivery	341	87.4	0.0000
Cesarean section	23	5.9	0.578
Instrumental delivery	26	6.7	0.9980
Presence of cultural reason for not to go for PNC services	84	21.5	0.001
Number of visits they know for PNC service			
Once	126	32.6	0.995
Two to four	54	13.8	0.994
Four and above	143	36.7	0.0000

### Postnatal care service utilization

**Fig. 2:**
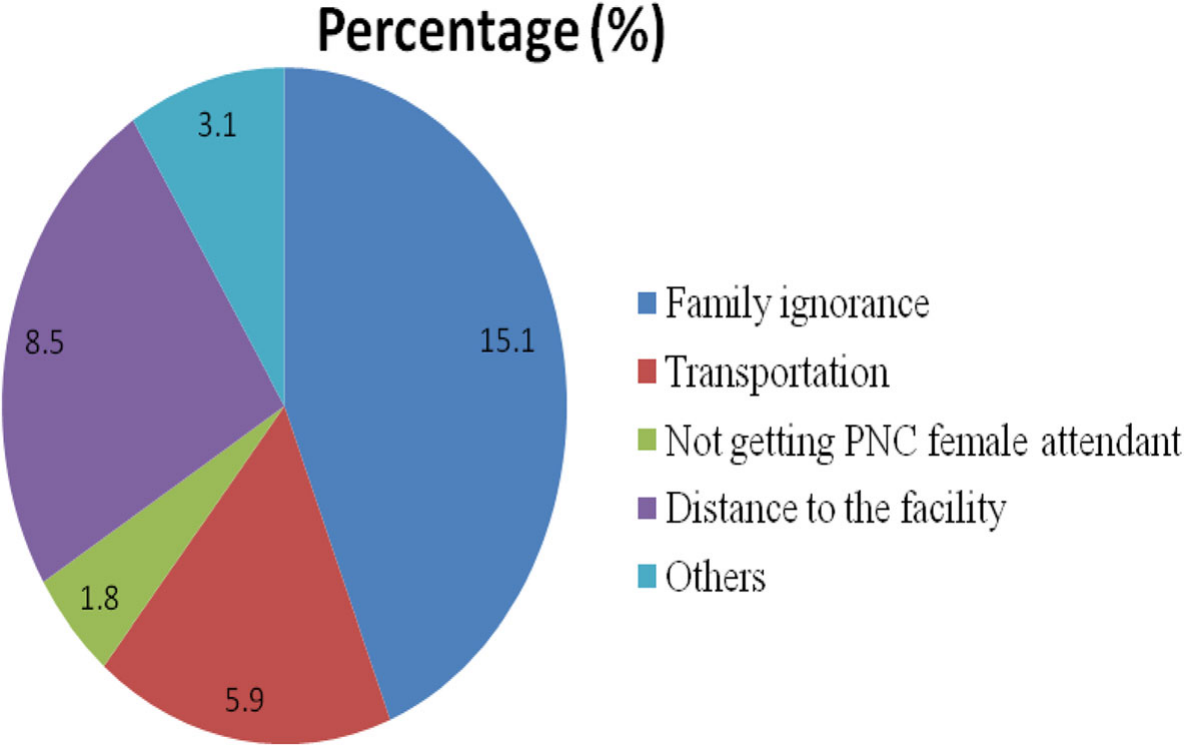
Reasons for non-utilization of PNC in Debre Birhan Town

Out of 390 women included in the study, 327 (83.8%) had utilized postnatal care service in the last 1 year of which 143 (36.7%) reported to have three or more postnatal visits at the time of the interview. Respondents gave different reasons for not getting a PNC visit. Among the several reasons given, 59 (15.1%) mentioned family ignorance (Fig. 2).

**Fig. 3:**
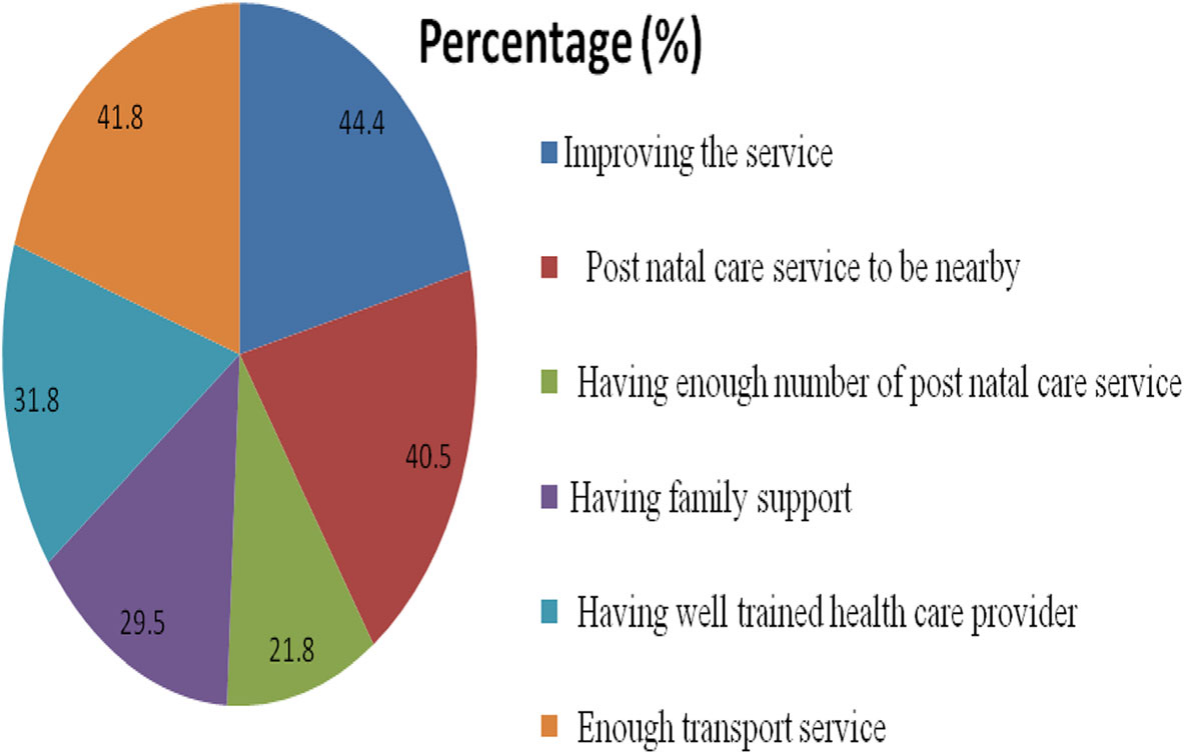
Participants suggestion for improving the PNC service in Debre Birhan town

Nearly half (173, 44.4%) suggest the service must be improved, 158 (40.55) responded the PNC service must be nearby, and 163 (41.8%) transport service must be improved to increase the utilization of PNC (Fig. 3).

### Socio-demographic, environmental, and clinical factors

Marital status, knowledge about PNC services, and place of birth has a significant association with PNC utilization. According to this study, a single mother was 0.06 times less likely to use PNC service than a married one [adjusted odds ratio (AOR) = 0.06, 95% CI (0.01, 0.45)] (Table 4).

**Table 4:** Socio-demographic factors associated with postnatal care utilization in Debre Birhan town, North Shoa, Ethiopia 2015 (*n* = 390)

Variables	PNC utilization		Crude OR	Adjusted OR
	No	Yes	(95% CI)	(95% CI)
Maternal age				
< 18	12(13.3)	78(86.7)	1	
18–24	16(11.5)	123(88.5)	1.18(0.53, 2.63)	
25–34	20(20.8)	78(79.6)	0.60(0.27, 1.31)	
≥ 35	15(23.8)	48(76.2)	0.49(0.21, 1.14)	
Marital status				
Married	45(13.7)	283(86.3)	1	1
Single	10(32.3)	21(67.7)	0.33(0.15, 0.75)	0.06(0.01, 0.45)
Divorced	5(20.8)	19(79.2)	0.60(0.21, 1.70)	0.20(0.03, 1.20)
Widowed	3(42.9)	4(57.1)	0.21(0.06, 0.98)	0.38(0.02, 8.04)
Maternal education				
Illiterate	19(42.2)	26(57.8)	1	
Grades 1–8	38(23.5)	124(76.5)	2.38(1.19, 4.77)	
Grades 9–12	6(4.9)	116(95.1)	14.1(5.13, 38.8)	
12+	0(.0)	61(100)	1.18(0.00)	
Husband’s education				
Illiterate	10(62.5)	6(37.5)	1	
Grades 1–8	35(29.4)	84(70.6)	4.00(1.35, 1.85)	
Grades 9–12	16(10.5)	136(89.5)	14.1(4.54, 44.1)	
12+	2(1.9)	101(98.1)	84.2(14.9473)	
Monthly income				
< 500	37(34.3)	71(75.7)	1	
500–1500	22(10.9)	179(89.1)	4.24(2.34, 7.69)	
1500–2500	4(6.7)	56(93.3)	7.30(2.45, 21.68)	
> 2500	0(0.0)	21(100.0)	8.41(0.00)	

### Obstetric factors

A significant association was found between the place of birth and PNC utilization. Mothers having institutional delivery were 0.65 times were more likely to utilize PNC services than those mothers who delivered at home [AOR = 0.65, 95% CI (0.58, 0.94)] (Table 5).

**Table 5:** Obstetric factors associated with PNC utilization among mothers in Debre Birhan town, North Shoa, Ethiopia, 2015 (*n* = 390)

Variables	PNC utilization		Crude OR	Adjusted OR
	No	Yes	(95% CI)	(95% CI)
Age at 1st pregnancy				
< 18	6 (26.1%)	17 (73.9%)	1	
18–35	56 (11.3%)	309 (84.7%)	1.947(0.74, 5.15)	
35 and above	1 (50%)	1 (50%)	0.353(0.019, 6.57)	
Gravidity			1	
1	19(12.3%)	135(87.7%)	0.7(0.39, 1.28)	
2–4	36(18.7%)	179(83.3%)	0.229(0.084, 0.62)	
≥ 5	8(38.1%)	13(61.9%)		
Number of live births				
1	19(11.7%)	143(88.3%)	1	
2–4	38(18%)	173(82%)	0.605(0.34, 1.09)	
≥ 5	6(31.3%)	11(64.7%)	0.24(0.08, 0.73)	
Ever had abortion				
Yes	1(7.1%)	14(92.9%)	2.57(0.33, 20.04)	
No	62(16.5%)	313(83.5%)	1	
Ever had still birth				
Yes	4(40.0%)	6(60%)	0.276(0.075, 1.007)	
No	59(15.5%)	321(84.5%)	1	
Ever had neonatal death				
Yes	59(15.6%)	319(84.4%)	2.703(0.79, 9.27)	
No	4(33.3%)	8(66.7%)	1	
Place of birth				
Home	11(30.6%)	25(69.4%)	1	1
Health facility	316(89.3%)	38(10.7%)	18.90(8.62, 41.4)	0.65(0.58, 0.94)

### Knowledge of mothers and PNC

Mothers’ knowledge have shown a significant association with utilization of PNC [AOR = 0.03, 95% CI (0.00, 0.44)] (Table 6).

**Table 6:** Knowledge-associated factors and PNC services among mothers in Debre Birhan Town, North Ethiopia, 2015 (*n* = 390)

Variables	PNC utilization		Crude OR	Adjusted OR
	Yes	No	(95% CI)	(95% CI)
Heard about PNC service	322(94.4%)	19(5.6%)	0.007(0.002–0.019)	
Knowledge of the existence of PNC service	321(95.3%)	16(4.7%)	0.005(0.002–0.014)	
Knowledge on the danger signs during postnatal period	322(95.5%)	15(4.5%)	0.005(0.002–0.014)	
Knowledge on the PNC services				
Yes	317(96.9%)	10(3.1%)	1	1
No	6(9)	57(90.5)	0.003(0.001, 0.009)	0.03(0.00, 0.44)
Distance from nearby health institution (meter)				
< 500	82(89.1%)	10(10.9%)	1	
501–2000	196(89.5%)	23(10.5%)	1.039(0.474–2.228)	
> 2001	49 (62%)	30(38%)	0.199 (0.09–0.443)	
ANC follow-up	320 (89.4%)	38(10.6)	0.033(0.013–0.082)	

## Discussion

In this study, it was learned that close to 84% of women sought at least one PNC from health institutions. This was consistent with a study done in Tigray in Adwa which was 78.3% [15] and another study done in Zimbabwe, 89.9% [20]. Our finding is contrary to much lower findings as evidenced by 66.83% [21], 51.4% [22], 38.2% [23], 34.8% [24], and 33.5% [25] PNC utilization in Amhara region. The reason for the high level of utilization of maternal health services among urban women in our study compared with their rural counterparts is that in most sub-Saharan countries, urban women in Ethiopia tend to benefit from increased knowledge and access to maternal health services. Additionally, health facilities are more accessible in urban areas and the various health promotion programs that use urban-focused mass media work to the advantage of urban residents in close connection to the use of maternal health services [26].

In line with the hypothesis, the PNC service utilization rate in Debre Birhan town was higher than the 2011 national EDHS report. Thus, the town serves as an exemplary site for postnatal health care service provision. The high rate could be due to the implementation of diverse maternal health care service intervention initiatives designed to improve access. The increased utilization rate could also be due to a homogeneous nature of study participants as compared with diverse study subjects in the 2011 EDHS. Among the socio-demographic variables, marital status was the only variable which has a significant association with utilization of postnatal care. Accordingly, mothers who were single are less likely to utilize postnatal services than mothers who are married and live together with their husbands. Similarly, a study conducted in another part of the country reveals that married women are more likely to receive postnatal care services than unmarried women [27]. Married women who live together with their husbands could be supported by their husbands than single women.

This study revealed that a respondent’s knowledge of postnatal care services is an important predictor of PNC utilization; accordingly, mothers who have knowledge of PNC services are more likely to utilize PNC than mother’s who do not have knowledge on PNC services. This finding was consistent with the study done in Uganda that revealed among those who were aware of the PNC services, a large proportion of mothers utilized the services [28].

Women who had awareness/knowledge of the postnatal services did utilize the PNC service two times higher than those women who were not adequately informed about the PNC service. Similarly, a study done in northern Ethiopia found that those women who had got information about postnatal care services utilizes PNC and were more likely to attend a postnatal care service compared to those women who had got no information [14]. This finding may lead to a conclusion that the PNC service utilization is strongly influenced by the knowledge of women on postnatal care benefits.

According to this finding, the other significant variable which has an influence on utilization of PNC among the obstetric variables is place of delivery in which PNC services are found to be more likely utilized among mothers who gave birth at a health facility than mothers who gave birth other than a health facility. Similar findings were also reported in North Western Ethiopia [14, 22, 25] and elsewhere [20, 28, 29].

### Strength and limitation of the study

The finding of the study was sent to the Amhara health bureau and ministry of health that encourage the stake holders for interventional measurements on the awareness with the involvement of one of the authors by giving health education and training for health professionals and women in the community. The study design was a cross-sectional which measures the exposure and outcome at the same time rather than a longitudinal design, so it is difficult to determine causal relationships between the proposed predictors and the outcomes of interest.

## Conclusion

The magnitude of PNC service utilization in Debre Birhan town was higher when it is compared with EDHS data. Marital status, knowledge of the mothers on PNC service, and place of delivery were determinants of postnatal care service utilization. So priorities have to be given to maternal health services especially institutional delivery, since place of delivery is the major determinant of postnatal care utilization. Particular attention has to be given for women’s education on health care issues; it is also recommended that better effort should be exerted to increase PNC utilization, by educating, motivating the public, and giving particular attention to mothers who are unmarried, divorced, and widowed.

## Abbreviations

AOR: Adjusted odds ratio
EDHS: Ethiopian demographic health survey
MDG: Millennium development goal
MMR: Maternal mortality rate
PNC: Postnatal care
SPSS: Statistical package for social science
WHO: World health organization

## References

[1] WHO (1998). Postpartum care of the mother and newborn: a practical guide WHO/RHT/MSM/983.

[2] Dhakal S, Chapman GN, Simkhada PP, Van ER, Stephens TJ, Raja AE. Utilization of postnatal care among rural women in Nepal. BMC Pregnancy Childbirth. 2007;7(19) 10.1186/1471-2393-7-19. 10.1186/1471-2393-7-19 PMC2075509 17767710

[3] Babalola S, Fatusi A (2009). Determinants of use of maternal health services in Nigeria—looking beyond individual and household factors. BMC Pregnancy Childbirth.

[4] Lawn J, Kerber K (2006). Opportunity for Africa newborns: practical data, policy and programmatic support for newborn care in Africa.

[5] Kaso M, Addisse M (2014). Birth preparedness and complication readiness in Robe Woreda, Arsi Zone, Oromia Region, Central Ethiopia: a cross-sectional study. Reprod Health.

[6] Alkema L, Chou D, Hogan D, Zhang S, Moller AB, Gemmill A (2016). Global, regional, and national levels and trends in maternal mortality between 1990 and 2015, with scenario-based projections to 2030: a systematic analysis by the UN Maternal Mortality Estimation Inter-Agency Group. Lancet.

[7] Rahman M, Haque SE, Zahan S (2011). Factors affecting the utilization of postpartum care among young mothers in Bangladesh. Health Soc Care Community.

[8] Central Statistical Agency [Ethiopia] and ICF International Ethiopia (2012). Demographic and Health Survey 2011.

[9] Wang W, Soumya A, Shanxiao W, Alfredo F (2011). Levels and trends in the use of maternal health Services in Developing Countries. DHS Comparative Reports no. 26.

[10] Oluwaseyi SD (2014). Determinants of postnatal care non-utilization among women in Nigeria.

[11] Li XF, Fortney JA, Kotelchuck M, Glover LH (1996). The postpartum period: the key to maternal mortality. Int J Gynaecol Obstet.

[12] DiBari JN, Yu SM, Chao SM, Michael CL (2014). Use of postpartum care: predictors and barriers. J Pregnancy.

[13] Jat TR, Nawi N, Sebastian MS. Factors affecting the use of maternal health services in Madhya Pradesh state of India. Int J Equity Health. 2011;10(59) 10.1186/1475-9276-10-59. 10.1186/1475-9276-10-59 PMC3283453 22142036

[14] Workineh YG, Hailu DA (2014). Factors affecting utilization of postnatal care service in Jabitena district, Amhara region, Ethiopia. Sci J Public Health.

[15] Berhe H, Tilahun W, Aregay A, Bruh G, Gebremedhim H (2013). Utilisation and associated factors of postnatal care in Adwa Town, Tigray, Ethiopia—a cross sectional study. Adv Res Pharmaceuticals Biologicals.

[16] Worku AG, Yalew AW, Afework MF. Factors affecting utilization of skilled maternal care in Northwest Ethiopia: a multilevel analysis. BMC Int Health Human Rights. 2013;13(1) 10.1186/1472-698x-13-20. 10.1186/1472-698X-13-20 PMC3639034 23587369

[17] Dagne E (2010). Role of socio-demographic factors on utilization of maternal health care services in Ethiopia.

[18] Ayele DZ, Belayihun B, Teji K, Ayana DA. Factors affecting utilization of maternal health care services in Kombolcha District, Eastern Hararghe Zone, Oromia Regional State, Eastern Ethiopia. Int Sch Res Not. 2014;(917058):1–8. 10.1155/2014/917058 PMC4897107 27437510

[19] Stella B, Adesegun F (2005). Determinants of use of maternal health services in Nigeria-looking beyond individual and household factors. BMC Pregnancy Childbirth.

[20] Innocent H, Seter S (1999). Prevalence and associated factors for non utilization of post natal care service population based study in Kuwadzana peri-urban area, Zvimba, District of Mashonland West Province, Zimbabwe. Afr J Reprod Health.

[21] Tesfahun F, Worku W, Mazengiya F, Kifle M (2014). Knowledge, perception and utilization of postnatal care of mothers in Gondar Zuria District, Ethiopia: a cross-sectional study. Matern Child Health J.

[22] Belachew T, Taye A, Belachew T (2016). Postnatal care service utilization and associated factors among mothers in Lemo Woreda, Ethiopia. J Women’s Health Care.

[23] Abuhay M. Assessment of factors influencing utilization of postnatal care in Gondar Town North West of Ethiopia. Thesis. 2008;1–98. etd.aau.etd. institutional repository.

[24] Hordofa MA, Almaw SS, Berhanu MG, Lemiso HB (2015). Postnatal care service utilization and associated factors among women in Dembecha District, Northwest Ethiopia. Sci J Public Health.

[25] Postnatal care service utilization and associated factors among women who gave birth in the last 12 months prior to the study in Debre Markos Town, Northwestern Ethiopia: a community-based cross-sectional study. Int J Reprod Med. 2016;2016. 10.1155/2016/7095352. 10.1155/2016/7095352 PMC4940555 27433481

[26] Mekonnen Y, Mekonnen A (2002). Utilization of maternal health care services in Ethiopia.

[27] Prashant S, Rajesh R, Manoj A, Lucky S (2012). Determinants of maternity care services utilization among married adolescents in rural India. International Institute for Population Sciences. PLoS One.

[28] Aminah K (2009). Factors determining utilization of postpartum care service in Uganda. hdi5.

[29] Nankwanga a (2004). Factors influencing utilization of postnatal services in Mulago and Mengo hospitals Kampala, Uganda.

